# Implementing an Interdisciplinary Procedure Curriculum

**DOI:** 10.7759/cureus.44851

**Published:** 2023-09-07

**Authors:** Bryce Montane, Abey Abraham, Sergio Bustamante, Tushar Vachharajani, Sabry Ayad, Jagan Devarajan, Dustin Thompson, Ran Lee, Penelope Rampersad, Brian Gage, Emily Reznicek, Chongliang Luo, Richard Wardrop

**Affiliations:** 1 Internal Medicine, Washington University School of Medicine, St. Louis, USA; 2 Cardiothoracic Anesthesiology, Cleveland Clinic, Cleveland, USA; 3 Nephrology, John D. Dingell Department of Veterans Affairs Medical Center, Detroit, USA; 4 Outcomes Research, Cleveland Clinic, Cleveland, USA; 5 Anesthesiology, Cleveland Clinic, Cleveland, USA; 6 Diagnostic Radiology, Cleveland Clinic, Cleveland, USA; 7 Cardiovascular Medicine, Cleveland Clinic, Cleveland, USA; 8 Gastroenterology, University of Missouri, Columbia, USA; 9 Surgery, Washington University School of Medicine, St. Louis, USA; 10 Internal Medicine, Cleveland Clinic, Cleveland, USA

**Keywords:** critical care and internal medicine education, critical, academic cardiology, interventional nephrology, nephrology, interventional radiology, anesthesiology, internal medicine, procedure training, interdisciplinary cooperation

## Abstract

Introduction: This curriculum was designed to improve access to procedures for our internal medicine residents.

Methods: We created an interdisciplinary procedure course (IDPC) composed of two simulation sessions and a one-week procedural rotation supervised by multiple specialties including nephrology, cardiology, cardiothoracic anesthesiology, general anesthesiology, and interventional radiology. After the course, residents completed two surveys documenting the number of procedures and their level of confidence on a Likert scale (1 = very unconfident to 5 = very confident) prior to and after completing the curriculum.

Results: Sixteen residents participated in the course from September 2021 to June 2022. The collective number of procedures performed by these 16 residents increased from 176 to 343 after a one-week rotation. For arterial lines, the proportion of residents that reported an improvement in confidence scores was 0.44 (95% confidence interval 0.23 to 1, p-value of 0.60). The proportion of residents that had an increase in their confidence performing central lines was 0.63 (95% confidence interval 0.39 to 1, p-value of 0.23). For intubations, the proportion of residents that reported an improvement in confidence was 0.94 (95% confidence interval 0.72 to 1, p-value of 0.0006).

Conclusion: By collaborating with multiple specialties, residents almost doubled the number of procedures performed during training and reported an increased level of confidence in procedural performance for airway intubation. We learned residents want to improve their access to procedures and described a curriculum that was easily implemented.

## Introduction

Internal medicine programs vary widely in how their trainees acquire the skills they need to perform medical procedures [1-13]. The old paradigm from Dr. William Halsted in 1890 for training surgical and procedural skills consisted of observing an intervention, performing one, and then teaching a trainee the skill [14]. This method of training has changed [1,7]. For example, a contemporary study about competency in central venous line placement recommended that each resident observe four, assist in four, perform four under supervision, and place five lines independently prior to graduating to assure competence [7].

Survey data conducted by Wigton et al. and the American College of Physicians (ACP) has shown the number of procedures performed by practicing general internists has declined dramatically [1,15]. This trend is attributed to a focus on patient safety, time constraints, and increased accessibility of other providers that routinely perform invasive procedures, and this trend affects residents, too [3,8,16-18]. The volume of cases normally available to residents has been shown to decrease with the installation of new fellowship programs and dedicated teams, which are meant to improve patient outcomes by utilizing providers with higher levels of experience [3,18,19]. However, sole reliance on a limited number of specialists to perform a procedure does come at a cost. Limited access to specialists can lead to procedural delays for patients and result in adverse outcomes [8,20]. Residency programs that provide an emphasis on procedural training correlate with success in resident recruitment [21]. Recently, institutions have shifted focus on procedural training via procedure rotations and resident-led initiatives [5,13]. We sought to expand on this idea to include faculty physicians who teach in different disciplines.

Many residents at our institution expressed a desire to learn essential procedural skills based on an internal needs assessment survey. We created a novel one-week interdisciplinary procedure course (IDPC) in conjunction with simulation learning to provide residents the opportunity to develop, practice, and polish their procedural skills. We chose this approach as interdisciplinary/interprofessional initiatives have shown to be very successful in the education of trainees [22-24]. To develop our IDPC, we implemented Kern’s six steps for curriculum development: problem identification, targeted needs assessment, goals and objectives, educational strategies, implementation, and evaluation and feedback [25].

## Materials and methods

Problem identification

As part of problem identification, we reviewed the recommendations from both the Accreditation Council for Graduate Medical Education (ACGME) and the American Board of Internal Medicine (ABIM) to understand the requirements for procedural training amongst internal medicine residents. Currently, ACGME and ABIM state that internal medicine programs must provide opportunities to learn common procedures [26,27]. Unfortunately, ACGME and ABIM no longer mandate a standardized method to assess competency or a prespecified number of common hospital procedures that each trainee must perform during residency. Without standardized requirements, procedural education and verification of competency vary [3,8,12,28].

Based on ACP’s survey results from 1986 to 1987, internal medicine residency program directors were asked how many of their graduates have mastered or should eventually master a particular procedural skill. Approximately 60%, 70%, and 40% of graduated residents were estimated to have mastered arterial line placement, central venous catheter (CVC) placement, and endotracheal (ET) intubation, respectively, during their training [1]. In comparison, 80%, 95%, and 85% of program directors surveyed reported that residents should master arterial line placement, CVC placement, and ET intubation, respectively [1]. When surveying general internists, it was found that from 1986 to 2004, the number of procedures they performed in the past year decreased from 27 to 8 arterial lines placed, 39 to 16 for CVC placed, and 41 to 15 for ET intubations performed [15]. The number of internists performing these procedures has continued to decline [8].

Goals and objectives

**Figure 1:**
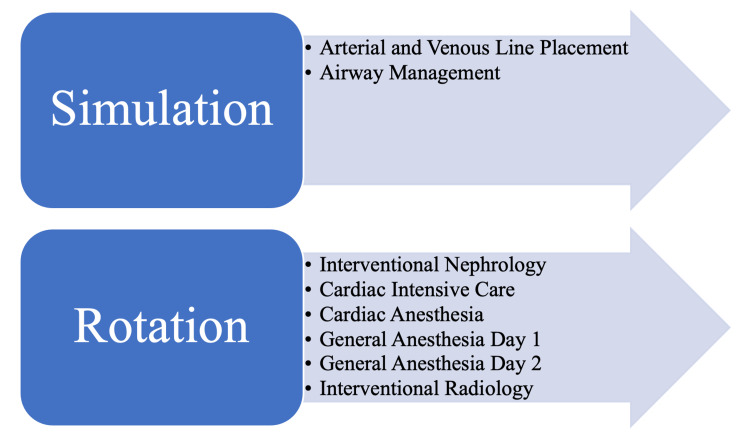
IDPC: simulation + rotation schedule This figure shows what was conducted in the simulation sessions and the departments for each rotation

Our goal was to improve procedural training in our residency program. The objectives of course included creating a unique curriculum composed of both simulated procedural sessions and hands-on experience during a clinical rotation (Figure 1). Recruitment of faculty and staff was done with a dedicated chief resident and our program director of internal medicine. Our goal was to have trainees rotate with multiple specialties: interventional nephrology, cardiac critical care, cardiac anesthesia, general anesthesia, and interventional radiology. There was no funding for this initiative; thus, all facilitators volunteered their time to teach our residents. We communicated with 22 stakeholders at four different hospitals to create a meaningful procedure course for our residents. Trainee procedural and internal survey data was provided to potential facilitators at our institution to galvanize support for the initiative. From this, we were able to recruit 10 physician leaders to instruct trainees at three of our hospital sites. These 10 physicians helped organize this course, provided expectations, and recruited additional providers for teaching purposes.

The simulated sessions provided the residents with foundational medical knowledge, proper safety standards, and practice prior to performing procedures on patients in a clinical setting. To maximize learning for each resident and to avoid over-commitment by facilitators, we allowed only one resident to rotate on the procedure course per week. We chose to have them rotate with a new specialty or faculty each day to allow for a variety of procedures to be performed within that week. The days of the week assigned to each specialty were selected based on the recommendation of each supervising staff to maximize the number of procedures available for training purposes.

Population selection

This course was approved as a quality improvement project with the exemption by our Institutional Review Board committee (study number 21-1142). This prospective study followed residents at our institution who agreed to participate in our pilot and was conducted from September 2021 to June 2022. The course was available to third-year internal medicine residents who volunteered and verbally consented to be part of this pilot course offered through email. We focused on third-year residents based on their stated desire to have more experience in performing procedures before entry into fellowship or unsupervised practice. Not all third-year residents, 55 in total, were interested in the course and our pilot could only be offered to 16 residents.

Recruitment of faculty to teach trainees was facilitated entirely by a chief resident through email, telephone, and face-to-face interactions. Each resident completed two prerequisite simulation sessions to maximize patient safety and procedural efficiency prior to a supervised weeklong procedural rotation with each facilitator [2,6]. Additionally, we were able to compare the number of procedures each resident performed during at least two and a half years of residency and, if applicable prior to residency, in comparison to our novel one-week interdisciplinary course. The number of residents allowed to participate in this pilot depended on the availability of their schedule and the accessibility for each supervising attending at each location.

Simulation sessions

With the interventional nephrologist, the residents received four hours of online training modules and a four-hour hands-on session. The four-hour online module reviewed the clinical relevance to central venous access, fundamentals of ultrasonography, familiarizing with procedural equipment, sonoanatomy, and common procedural complications. This module was followed by a video of the central venous line placement. The simulation lab allowed residents to review steps to practice central venous line placement safely. The trainee had to demonstrate proficiency at four stations. Station one consisted of learning the different modes of ultrasound technique, types of probes, and ergonomics of holding the device. Each trainee used ultrasound to identify the relevant anatomy of a colleague. They were asked to verbalize the correct positioning of the patient for central line placement and setting up the equipment and supplies. At station two, each trainee learned how to identify the needle tip under ultrasonography and advance the needle tip in the vessel lumen using the “tip tracking” method [17]. At station three, they learned the correct technique to scrub, gown, and glove. The fourth station consisted of performing a central line placement on a manikin.

The indication for line placement is followed by the consenting and time-out safety protocol for correct patient and procedure identification, proper patient positioning, and setting up equipment. Afterward, they verbalized post-procedure steps: ordering a chest X-ray and reviewing techniques to confirm tip positioning. Abnormal chest X-rays with malpositioned catheter tip and complications such as pneumothorax were reviewed. The simulation course ended with a short debriefing and reflection session to elicit thoughtful discussion.

Most of the trainees had no training in ET intubation during residency. While many institutions do not require internal medicine physicians to perform intubations, many residents asked we include it as part of the curriculum, especially those interested in a career in cardiac, pulmonary, or critical care [29]. The three-hour simulation session began with a lecture-style presentation where foundational knowledge and the objectives were outlined: understanding the basic approaches to airway management, recognizing risk factors and rescue steps for a difficult airway, identifying key anatomical landmarks, and steps needed for intubation.

After the lecture, residents underwent task training, a high-fidelity simulation, and ended with a debriefing session. Using deliberate practice and mastery learning principles, the internal medicine residents were able to practice bag-mask ventilation, placement of oral and nasopharyngeal airways, supraglottic airway placement, and oral intubation using direct and video laryngoscopy. The simulation lab emulated an operating room with an anesthesia machine and used a high-fidelity simulator in an induction of general anesthesia. The final element was a constructive debriefing session whereby we reflected and discussed the importance of calling for help early, along with consolidating key take-home points. During each simulation session, a maximum of four trainees were taught to maximize individual learning needs. Time was taken to ensure residents understood the indications of a procedure, contraindications to a particular procedure, proper sterile technique, and steps to avoid procedural complications.

Timing

Schedules were modified to accommodate the course with the help of the chief residents at our institution. January was chosen as the starting time of the one-week rotation so that resident learning could be prioritized over each specialty’s ACGME fellows who start on July 1 of each year. This time was also chosen to avoid conflicts with third-year residents who were interviewing for fellowship/positions in the Fall. The days of the week assigned to each specialty were selected based on the recommendation of each supervising staff to maximize the number of procedures available for training purposes. Even if no procedures were performed that day (i.e., no acute needs in the intensive care unit), the procedure day was still counted toward their total number of days so long as the resident and attending physician were present.

The procedure rotation

Residents rotated with each subspecialty (nephrology, cardiology, cardiothoracic anesthesiology, general anesthesiology, and interventional radiology) from January 2022 to June 2022. Residents performed procedures on patients receiving inpatient care at our institution and were supervised by attending physicians and advanced practice providers. All procedures performed were scheduled apart from those conducted in the cardiac intensive care unit. The weeklong procedure rotation included rotating with interventional nephrology (day 1), cardiac intensive care (day 2), a half day of cardiac anesthesia (day 3), general anesthesia (days 4 and 5), and interventional radiology (day 6). With this schedule, this meant we only required a commitment of 16 days from each faculty member. The schedule was novel as none of the participants had ever rotated with any of these specialties, apart from cardiac intensive care. These individuals recorded the number of and their level of confidence in performing the following procedures before and after this course: arterial lines, central lines, and airway intubations. These three procedures were chosen based on our internal assessment surveys from the residents. The number of procedures performed was confirmed with online procedural logs. The number of days attended during the procedure course was recorded (maximum of 5.5 days). It was expected that the course would have absences due to clinical conflicts.

Surveys/feedback

Each resident was asked how many arterial lines, central lines, and airway intubations they had performed prior to and after the course. The residents were also asked to rate their level of confidence in performing each procedure before and after the course using a Likert scale of 1 to 5 (1 = very unconfident and 5 = very confident). The survey can be seen in the Appendices section. Faculty from each specialty were met after the conclusion of the course to provide verbal feedback, challenges, and improvements for the future.

Statistics

Our course was evaluated using a retrospective pre- and post-assessment which have shown to reduce response-shift bias for self-assessments [30,31]. In these surveys, residents were asked to evaluate their level of confidence in performing procedures. Our type I (false positive) error rate was set at 0.05. This error was then adjusted for three separate comparisons using the Bonferroni method. The Bonferroni method adjusts p-values to avoid an increased risk of a type I error that occurs when multiple statistical tests are conducted on the same data set. The equation is adjusted type I error=p value/number of comparisons, and a p-value of 0.0167 was considered significant for this study. The outcome of interest was the mean proportion of residents who improved when comparing pre- and post-assessment confidence scores for each procedure. Our null hypothesis was the difference in pre- and post-assessment values would be less than or equal to 0 (<0.5 proportion of residents will improve). Success was defined as more than 50% of trainees (proportion of 0.5) showed improved confidence before and after the course. Proportions were calculated using the binomial theorem as the data was not assumed to have a normal distribution based on the small sample size. Ninety-five percent confidence intervals for the outcome of interest were calculated. Median, 25th percentile, 75th quartile, and interquartile range (IQR) scores for each domain in the pre- and post-assessments were calculated.

## Results

Targeted needs assessment

As part of our needs assessment, we reviewed our annual survey given to all internal medicine residents. Many individuals reported that they had little access to procedures during their training. We then reviewed the number of procedures performed by our trainees, which host 165 categorical internal medicine residents each year, using our online platform where residents electronically log their procedures. When looking at procedures completed from January 2018 to June 2021 (a total of 3.4 years), 300 arterial lines, 298 central lines, and 27 airway intubations were performed by residents. When averaged by year, a total of approximately 88 arterial lines, 88 central lines, and 8 intubations were performed annually by all the internal medicine residents at our institution combined.

For this reason, we chose to create a new avenue for residents to learn and perform procedures in a hospital setting. Thirty-three out of 55 third-year internal medicine residents expressed interest in changing their schedule to accommodate the new procedure course. Based on the flexibility of the residents’ schedules and the availability of supervising staff, 16 (29.1%) residents rotated as the pilot group of the IDPC.

Implementation

As required by the course, all 16 residents completed the airway and line placement simulation sessions in the months prior to their scheduled weeklong procedure rotation. Participants performed a median number (25th, 75th quartile, IQR) of 4.5 (2, 6.75, 4.75) arterial lines, 3 (2, 5, 3) central lines, and 0.0 (0, 0.75, 0.75) intubations prior to the course. After the IDPC, the median number of arterial lines, central lines, and ET intubations performed were 5.5 (2.25, 7.0, 4.75), 5.5 (3.0, 9.0, 6.0), and 8.0 (4.0, 12.0, 8.0), respectively. In total, our resident sample increased the number of procedures they performed collectively from 176 to 343 (Table 1). The median number (25th, 75th quartile, IQR) of days residents were able to participate in the IDPC was 4.5 (3.75, 5.5, 1.75) out of 5.5 days. The reasons for days missed included jeopardy redeployment, sick days, personal days, procedure cancellations due to weather (snowstorm), and cancellations by the attending physician due to conflicting responsibilities.

**Table 1:** Pre- and post-IDPC survey This table depicts the number of arterial lines, central lines, intubations, and total number of procedures performed by all 16 residents combined before and after the IDPC. Residents rated their confidence in performing each procedure before and after the IDPC on Likert scales (1 = very unconfident to 5 = very confident). The number of days each resident was able to attend (maximum 5.5 days) of the procedure course is shown

Participant	Arterial lines				Central lines				Intubations				Days attended
	Number		Confidence		Number		Confidence		Number		Confidence		
	Pre	Post	Pre	Post	Pre	Post	Pre	Post	Pre	Post	Pre	Post	
1	5	5	2	3	3	7	2	3	0	4	1	4	5.5
2	10	10	4	4	2	5	2	2	0	3	1	2	4.5
3	6	6	3	4	4	5	2	3	3	4	1	2	1.5
4	5	6	3	4	9	11	4	4	0	2	1	3	3.5
5	2	2	3	3	2	4	3	4	0	8	1	4	5.0
6	2	2	1	2	2	7	1	4	0	12	1	4	5.5
7	3	6	3	4	1	3	2	3	1	7	1	3	5.5
8	4	4	4	4	5	9	3	4	0	8	1	3	4.5
9	1	1	2	2	0	0	2	2	10	17	3	4	4.5
10	7	7	4	4	5	6	3	4	0	13	1	3	4.5
11	0	0	3	3	0	0	2	2	0	0	1	1	0.0
12	2	3	3	3	3	3	2	2	0	10	1	3	5.5
13	15	18	4	4	20	23	3	4	13	33	1	3	5.5
14	5	9	3	5	4	10	3	4	0	4	1	4	4.5
15	3	3	2	3	3	3	2	3	0	12	1	4	3.5
16	7	7	4	4	9	9	4	4	0	12	1	4	4.5

At baseline (5-point Likert scale), the median (25th, 75th quartile, IQR) confidence in performing arterial lines was 3.0 (2.25, 4.0, 1.75) for arterial lines, 2.0 (2.0, 3.0, 1.0) for central lines, and 1.0 (1.0, 1.0, 0.0) for intubations. After the IDPC, the median confidence for performing arterial lines, central lines, and ET intubations was 4.0 (3.0, 4.0, 1.0), 3.5 (2.25, 4.0), and 3.0 (3.0, 4.0, 1.0), respectively. The outcome of interest was the proportion of residents who improved when comparing pre- and post-assessment confidence scores. A proportion above 0.5 means a substantial improvement in confidence. The detailed survey result is in Table 1. For arterial lines, the proportion of residents who reported an improvement in confidence was 0.44 (7 out of 16, 95% confidence interval 0.23 to 1, p-value of 0.60). For central lines, the proportion of residents who reported an improvement in confidence was 0.63 (10 out of 16, 95% confidence interval 0.39 to 1, p-value of 0.23). For intubations, the proportion of residents who reported an improvement in confidence was 0.94 (15 out of 16, 95% confidence interval 0.72 to 1, p-value of 0.0006).

## Discussion

In this study, we sought to improve resident access to procedures by involving multiple medical disciplines. In this prospective study of 16 third-year internal medicine residents, residents performed 3.1 procedures per day during the one-week procedure rotation. This is significant as with the addition of an IDPC elective, which required two half days of simulation plus five and a half days of a procedure rotation, the total number of procedures ever performed by these residents increased nearly twofold. We also found the level of confidence in performing intubations also increased significantly.

Evaluation and feedback

During the IDPC, residents rotated at a different location each day. This provided insight into which departments provided more procedures to trainees. Based on the results, we found residents were able to dramatically increase the number of intubations they had performed after rotating with the anesthesia department, with an average number of fewer than two intubations performed per resident prior to the course to more than nine intubations per resident after the course. Based on verbal feedback from our residents, we believe this profound increase was due to the population being intubated being scheduled for these procedures, and, therefore, there was a surplus. They were able to perform in comparison to the ICU which has more variability in the number of procedures offered on a day-to-day basis. In addition, residents reported more success at our smaller community hospitals (250 to 500 hospital beds) than at the main campus (an academic medical center with >1300 hospital beds) where services had more time for each procedure. The feedback from the faculty was very positive as they felt having to commit to only 16 days over 6 months made it very feasible for them to supervise and teach residents.

We found that the self-assessed level of confidence in performing intubations increased after the course. We believe that working with real patients combined with the prerequisite simulation sessions led our residents to have a meaningful improvement in their confidence to perform these procedures [6,12,13]. By collaborating with supervising physicians from multiple disciplines, internal medicine residents can improve their access to key procedures in a way that is feasible for both trainees and facilitators [22,23].

Improvement for the future

From the feedback we received, we can improve the elective in the upcoming years. We believe expanding the project to include more rotations in our community hospitals, where there are fewer trainees and many scheduled procedures, would greatly increase procedural opportunities for our residents. In addition, now that we have shown success in increasing the number of procedures, we would like to formally improve upon trainee competence in procedural skills. Formative assessments by dedicated faculty would help ensure that we are also improving the quality of procedural skills and outcomes for our trainees. We hope to create the assessments with the mindset of providing feedback in person and demonstrate knowledge of a procedure in a checklist format and a global rating of procedural skill for each resident [32,33]. If we are able to garner more interest after this, we hope to add a team-based learning style where residents can teach one another, which could provide more facilitators and motivation for individuals to take advantage of procedural opportunities [32]. The last addition to the course, we would like to create a formal logbook after our formative evaluations that will provide a more holistic review of each trainee, and this logged information could be used for future career positions as a form of academic achievement [34].

Limitations

There were several limitations presented in this study. There was no formal control arm, as all 16 residents studied were enrolled in our procedure course. This course focused on expanding opportunities for only three procedures (arterial lines, central lines, and ET intubations). These procedures were chosen based on our annual survey given to all internal medicine residents. Other procedures relevant to the training of an internal medicine physician, for example, arthrocentesis and lumbar puncture, were not included due to the limited resources of this pilot.

Residents rotated at different sites every day to help understand where residents were able to get the most hands-on experience. This led to certain residents being unable to work at specific locations due to unexpected absences. In addition, the resources of the course were limited in terms of weeks available to residents and supervising staff. In the future, we hope to expand this course to offer it to more residents, create a steady stream of residents to rotate with these disciplines outside of traditional internal medicine courses, and allow residents to be able to rotate for longer periods of time based on their subspecialty interests. Residents reported more difficulty getting procedural experience while in the intensive care unit, where patients were sometimes unstable or were intubated with invasive lines upon arrival. Currently, the elective is still growing with plans to be offered this upcoming academic year.

## Conclusions

Internal medicine programs lack unified standards for procedural training. We found that internal medicine residents want to improve their procedural skills and that opportunities to learn have decreased compared to previous training standards. We created a course where internal medicine residents rotated with multiple disciplines: interventional nephrology, cardiac intensive care, general and cardiac anesthesiology, and interventional radiology. We found that a combination of simulation sessions and a procedure elective improved confidence levels in performing specific procedures, and residents were able to increase the number of procedures they performed within a single week. In this study, we describe a curriculum to teach residents procedures with both simulation and hands-on experience with a method that was easily implemented into their training. We hope other institutions will use our IDPC as a model to implement in their own programs.

## Appendices

Evaluation: pre- and post-IDPC course survey

(Answers to both surveys are to be filled out after finishing the course)

Number: 

How many arterial lines have you performed? 

Pre:                            Post:

How many central lines have you performed? 

Pre:                            Post:

How many endotracheal intubations have you performed? 

Pre:                            Post:

Confidence: 

Please describe your level of confidence in performing the following procedures on a Likert scale from 1 to 5 (1 = very unconfident and 5 = very confident)

Arterial line:

Pre:                            Post:

Central line:

Pre:                            Post:

Endotracheal intubation:

Pre:                            Post:
